# Association between estimation of pulse wave velocity and all-cause mortality in critically ill patients with non-traumatic subarachnoid hemorrhage: an analysis based on the MIMIC-IV database

**DOI:** 10.3389/fneur.2024.1451116

**Published:** 2024-08-01

**Authors:** Jianquan Li, Meimei Zhang, Baning Ye, Mingjie Lu, Gang Liao

**Affiliations:** ^1^Department of Critical Care Medicine, Guizhou Provincial People's Hospital, Guiyang, China; ^2^Department of Neonatology, Shanghai Children's Medical Center Guizhou Hospital, Guiyang, Guizhou, China

**Keywords:** non-traumatic subarachnoid hemorrhage, estimated pulse wave velocity, subgroup analysis, mortality, MIMIC-IV

## Abstract

**Background:**

Estimated pulse wave velocity (ePWV), which measures vascular aging, is an independent predictor of cardiovascular death. Nevertheless, the relationship between ePWV and all-cause mortality among patients suffering from non-traumatic subarachnoid hemorrhages (NSAH) remains obscure. Consequently, the objective of this study is to ascertain whether ePWV exerts influence on the prognosis of individuals afflicted with NSAH.

**Methods:**

Through the Medical Information Mart for Intensive Care IV (MIMIC-IV) database, 644 eligible participants were included. The Kaplan–Meier survival curve method was employed to assess the disparity in survival status between the low and high ePWV cohorts. The Cox proportional hazard model was employed to investigate the association between ePWV and inpatient mortality among critically ill patients diagnosed with NSAH. The Restricted Cubic Spline (RCS) model was employed to examine the dose–response correlation. Subsequently, multivariate Cox regression analysis was performed to identify independent prognostic factors. Lastly, the impact of ePWV on inpatient mortality across various subgroups was evaluated through stratified analysis.

**Results:**

Participants were categorized into two groups, delineated by their ePWV levels: a low ePWV level group and a high ePWV level group. Survival analysis unveiled that individuals with high ePWV exhibited a diminished survival rate compared to their counterparts with low ePWV. Following adjustment, low ePWV was significantly linked with a reduced risk of inpatient mortality among patients with NSAH (HR = 0.54, 95% CI = 0.32–0.89, *p* = 0.016). Simultaneously, analysis employing the RCS model further substantiated a linear escalation in the risk of inpatient mortality with increasing ePWV values.

**Conclusion:**

Elevated ePWV levels have been identified as an independent risk factor for the rise in inpatient mortality among NSAH patients and as a significant predictor of the clinical outcome of NSAH.

## Introduction

NSAH denotes a clinical manifestation wherein diseased blood vessels located at the base or surface of the brain rupture, allowing blood to directly flow into the subarachnoid space, often associated with aneurysmal rupture (1). The mortality rate associated with NSAH is exceedingly high. Research indicates that almost a quarter of patients experience sudden death either en route to the hospital or in the emergency room (2, 3). Furthermore, another study reveals that among hospitalized individuals, the 30-day mortality rate for NSAH patients reaches as high as 35% (4). Additionally, some survivors of NSAH may experience physical disabilities and neuropsychological complications (3).Consequently, there is a pressing need for a reliable and readily available clinical index to ascertain prognosis.

The ePWV can be directly calculated based on actual age and average arterial blood pressure. As a marker of vascular aging, ePWV has increasingly emerged as a predictor of cardiovascular diseases and all-cause mortality in recent years (5, 6). Previous studies have established correlations between ePWV and certain diseases, including heightened all-cause mortality in coronary artery disease and acute kidney injury, as well as an increased risk of diabetes and stroke (7–10). While NSAH can result from head trauma, the majority is caused by abnormal intracranial vascular rupture (11). Nevertheless, it remains unclear whether ePWV levels are associated with variations in NSAH mortality risk. Thus, the aim of this study is to investigate the relationship between ePWV and NSAH using an open clinical database.

## Materials and methods

### Data sources and setting

The retrospective cohort analysis utilized the MIMIC-IV version 2.2 database (12, 13). This study is a longitudinal single-center investigation. During the period spanning from 2008 to 2019, a total of 299,712 patients were included, with 73,181 admitted to the intensive care unit. The author of this study, Gang Liao (ID: 12855703), registered, completed the required training, and obtained permission to utilize the dataset. As the MIMIC-IV database is openly accessible and anonymized, it does not necessitate approval from the ethics committee.

### Study population

**Figure 1:**
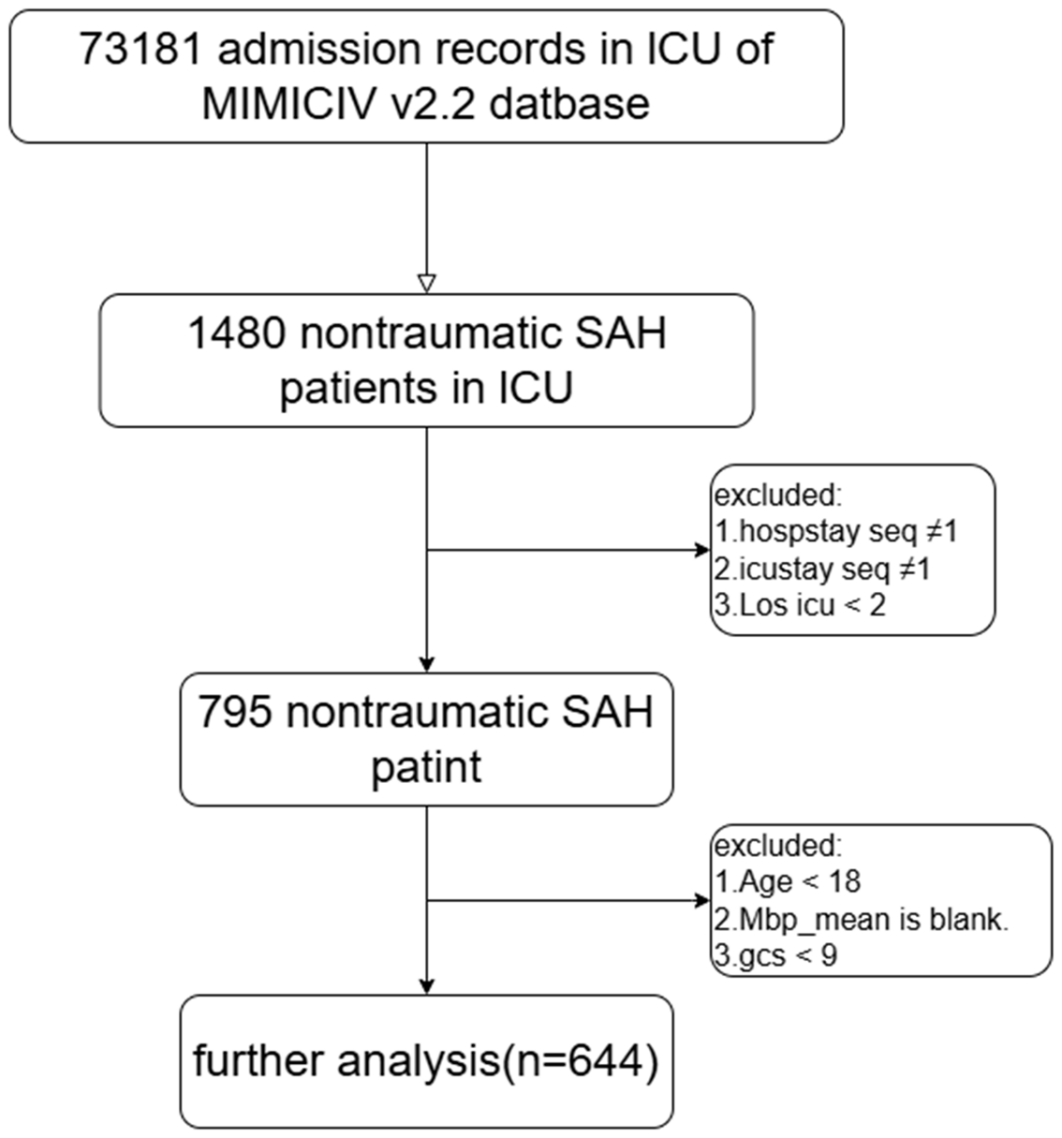
The flowchart of patients’ selection.

The study included adult NSAH patients classified based on the ninth and tenth revisions of the International Classification of Diseases (ICD-9 and ICD-10), along with admission codes for acute physiology and chronic health evaluation. Exclusion criteria encompassed patients meeting the following conditions: (1) multiple hospitalizations or ICU admissions, (2) ICU stays of less than 2 days, (3) age under 18 years, (4) lack of MAP information, and (5) GCS score less than 9. Ultimately, the study cohort comprised 644 patients, divided into two groups based on their median ePWV at ICU admission (Figure 1).

### MAP and ePWV measurement

Mean arterial pressure (MAP) was calculated using the formula: MAP = DBP + 0.4 × (SBP – DBP) (14). The formula for calculating ePWV is as follows: ePWV = 9.587–0.402 × age + 4.560 × 10^(−3) × age^2–2.621 × 10^(−5) × age^2 × MAP +3.176 × 10^(−3) × age × MAP – 1.832 × 10^(−2) × MAP (15).

### General data collection and outcomes

Data extraction was performed using Navicat 16 for PostgreSQL software. The extracted variables comprised: (1) Registration data: gender and age; (2) Complications: myocardial infarction (MI), congestive heart failure (CHF), anemia, diabetes, chronic obstructive pulmonary disease (COPD), and acute kidney injury (AKI); (3) Vital signs: Mean arterial pressure (MAP), resp_rate, temperature, spo2 and heart rate; (4) Laboratory tests: glucose, bicarbonate, and aniongap; (5) Disease severity scores: simplified acute physiology score ii (SAPS II), logistic organ dysfunction system (LODS), and glasgow coma scale (GCS). The final outcome of the study was the mortality rate during hospitalization. Moreover, insufficient samples of mechanical ventilation and vasoactive medications were gathered, resulting in their exclusion from the study.

### Statistical analysis

Baseline patient characteristics are described using the mean and standard deviation for continuous variables, and frequency and percentage for categorical variables. Differences in baseline characteristics are assessed using either a *t*-test or Mann–Whitney *U*-test, depending on the data distribution. Survival time for deceased patients is calculated from their ICU stay. Conversely, for patients who survived after discharge, their last recorded survival time was substituted with the longest survival time observed among deceased patients. Moreover, based on the optimal critical values of glucose, SAPS II score, and LODS score determined using X-tile software, these three parameters were dichotomized into high and low categories.

Kaplan–Meier survival curves were utilized to assess survival rates between the low and high ePWV groups, with between-group differences assessed using the log-rank test. Covariates were adjusted using the Cox proportional hazards model. Model 1 included adjustments for gender and age, while model 2 included adjustments for age, gender, myocardial infarction, congestive heart failure, anemia, diabetes, chronic obstructive pulmonary disease, and acute kidney injury. The restricted cubic spline (RCS) model was employed to assess the presence of a dose–response relationship between ePWV and all-cause mortality among NSAH patients. A likelihood ratio test was performed to assess for potential nonlinearity. Independent prognostic factors for NSAH patients were identified using multivariate Cox regression analysis. Subsequently, these factors were stratified to assess potential differences in the influence of ePWV on all-cause mortality across subgroups. The likelihood ratio test was employed to evaluate interactions between ePWV and stratification variables. Results were reported as hazard ratios (HRs) with corresponding 95% confidence intervals (CI). All statistical analyses were conducted using R software (version 4.2.0). All tests were two-tailed, with statistical significance set at *p* < 0.05.

## Results

### Patient characteristics

In the final analysis, a total of 644 adult patients with non-traumatic subarachnoid hemorrhage were collected from the MIMIC-IV v2.2 database. Baseline patient characteristics were compared between the low ePWV group (ePWV ≤8.728 m/s, 322 subjects) and high ePWV group (ePWV >8.728 m/s, 322 subjects) (Table 1). The mean ePWV for the two groups was 7.22 m/s and 10.8 m/s, respectively. Patients with high ePWV usually show older age and higher blood sugar, and have a higher probability of acute myocardial infarction, congestive heart failure, diabetes, anemia, chronic obstructive pulmonary disease and acute kidney injury. Additionally, the duration of ICU treatment was lower in the high ePWV group (LOS_ICU = 7.54 days) compared to the low ePWV group (LOS_ICU = 7.00 days), with a concurrent increase in all-cause mortality (25.8% versus 13.7%).

**Table 1:** Baseline characteristics of NSAH patients grouped according to ePWV.

	[ALL]	High	Low	p.overall
	*N* = 644	*N* = 322	*N* = 322	
Age				<0.001
18–50	158 (24.5%)	0 (0.00%)	158 (49.1%)	
50+	486 (75.5%)	322 (100%)	164 (50.9%)	
Gender				0.693
Female	348 (54.0%)	177 (55.0%)	171 (53.1%)	
Male	296 (46.0%)	145 (45.0%)	151 (46.9%)	
Heart_rate	64.0 [56.0,73.0]	63.0 [56.0,70.0]	64.0 [57.0,75.0]	0.018
MAP	81.8 [75.8,87.9]	81.8 [75.4,88.4]	81.7 [75.9,87.6]	0.630
Resp_rate	17.6 [16.0,19.8]	17.6 [16.1,19.9]	17.6 [15.9,19.6]	0.718
Temperature	37.0 [36.8,37.3]	37.0 [36.7,37.3]	37.0 [36.8,37.3]	0.063
spo2	97.8 [96.3,98.9]	97.5 [96.1,98.9]	98.0 [96.4,99.1]	0.030
MI				0.009
No	586 (91.0%)	283 (87.9%)	303 (94.1%)	
Yes	58 (9.01%)	39 (12.1%)	19 (5.90%)	
CHF				<0.001
No	596 (92.5%)	285 (88.5%)	311 (96.6%)	
Yes	48 (7.45%)	37 (11.5%)	11 (3.42%)	
Anemia				0.030
No	515 (80.0%)	246 (76.4%)	269 (83.5%)	
Yes	129 (20.0%)	76 (23.6%)	53 (16.5%)	
Diabetes				0.037
No	629 (97.7%)	310 (96.3%)	319 (99.1%)	
Yes	15 (2.33%)	12 (3.73%)	3 (0.93%)	
COPD				0.007
No	620 (96.3%)	303 (94.1%)	317 (98.4%)	
Yes	24 (3.73%)	19 (5.90%)	5 (1.55%)	
AKI				0.242
No	578 (89.8%)	284 (88.2%)	294 (91.3%)	
Yes	66 (10.2%)	38 (11.8%)	28 (8.70%)	
Aniongap	15.0 [14.0,18.0]	15.0 [14.0,18.0]	15.0 [13.0,18.0]	0.247
Bicarbonate	23.0 [21.0,25.0]	23.0 [21.0,26.0]	22.0 [20.0,25.0]	<0.001
Glucose				0.028
148+	188 (29.2%)	107 (33.3%)	81 (25.2%)	
40–148	455 (70.8%)	214 (66.7%)	241 (74.8%)	
Sapsii				<0.001
32+	296 (46.0%)	201 (62.4%)	95 (29.5%)	
6–32	348 (54.0%)	121 (37.6%)	227 (70.5%)	
LODS				0.603
0–5	532 (82.6%)	263 (81.7%)	269 (83.5%)	
5+	112 (17.4%)	59 (18.3%)	53 (16.5%)	
GCS				0.003
12–15	621 (96.4%)	303 (94.1%)	318 (98.8%)	
9–12	23 (3.57%)	19 (5.90%)	4 (1.24%)	
ePWV	8.73 [7.22,10.7]	10.8 [9.47,12.6]	7.22 [6.49,7.95]	<0.001
los_icu	7.33 [3.86,12.8]	7.00 [3.71,12.9]	7.54 [4.09,12.7]	0.323
Status				<0.001
Alive	517 (80.3%)	239 (74.2%)	278 (86.3%)	
Dead	127 (19.7%)	83 (25.8%)	44 (13.7%)	

### The relationship between ePWV and outcome

**Figure 2:**
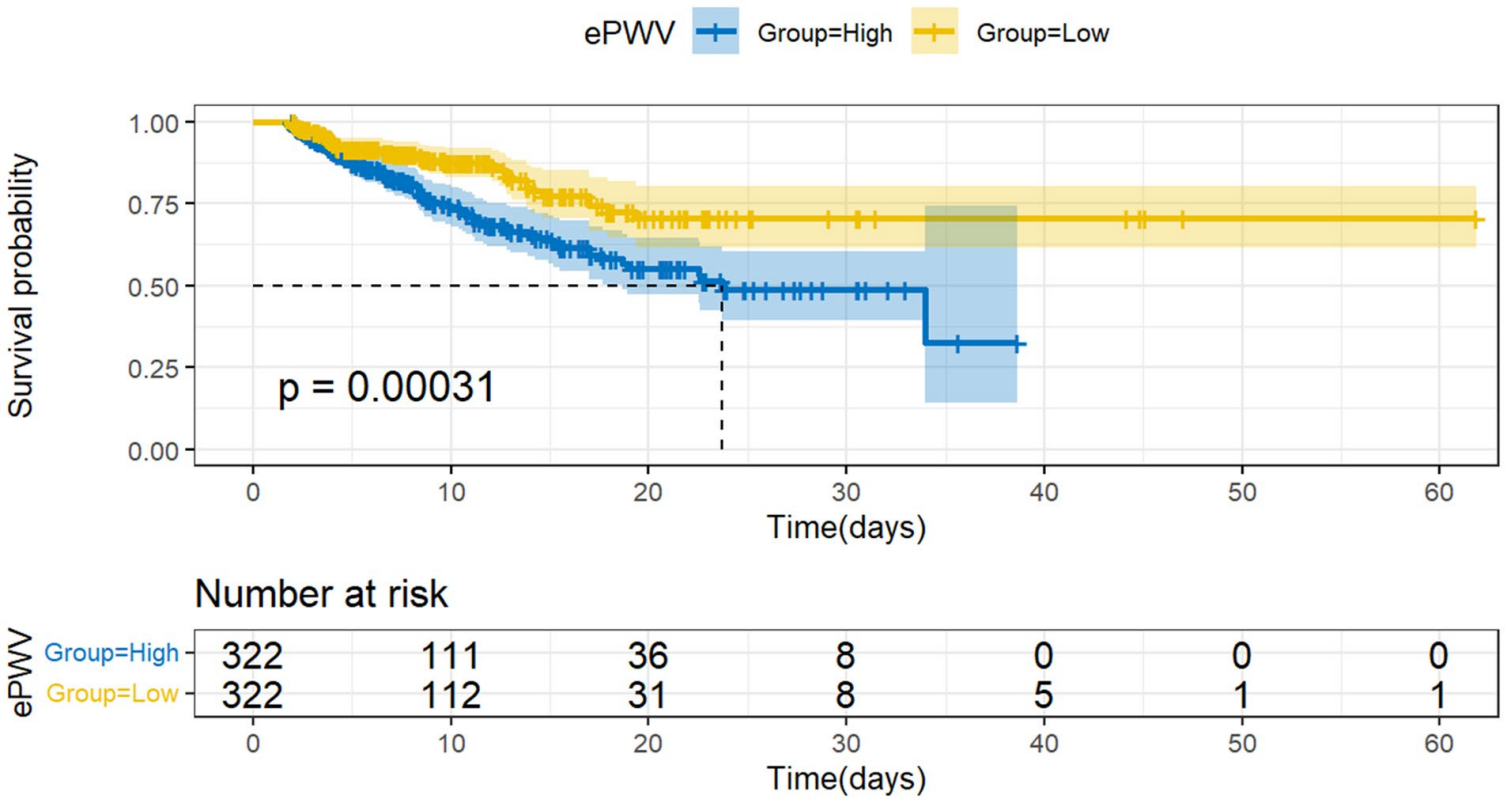
Kaplan–Meier survival curves for al-cause mortality among low ePWV group and high ePWV group.

**Figure 3:**
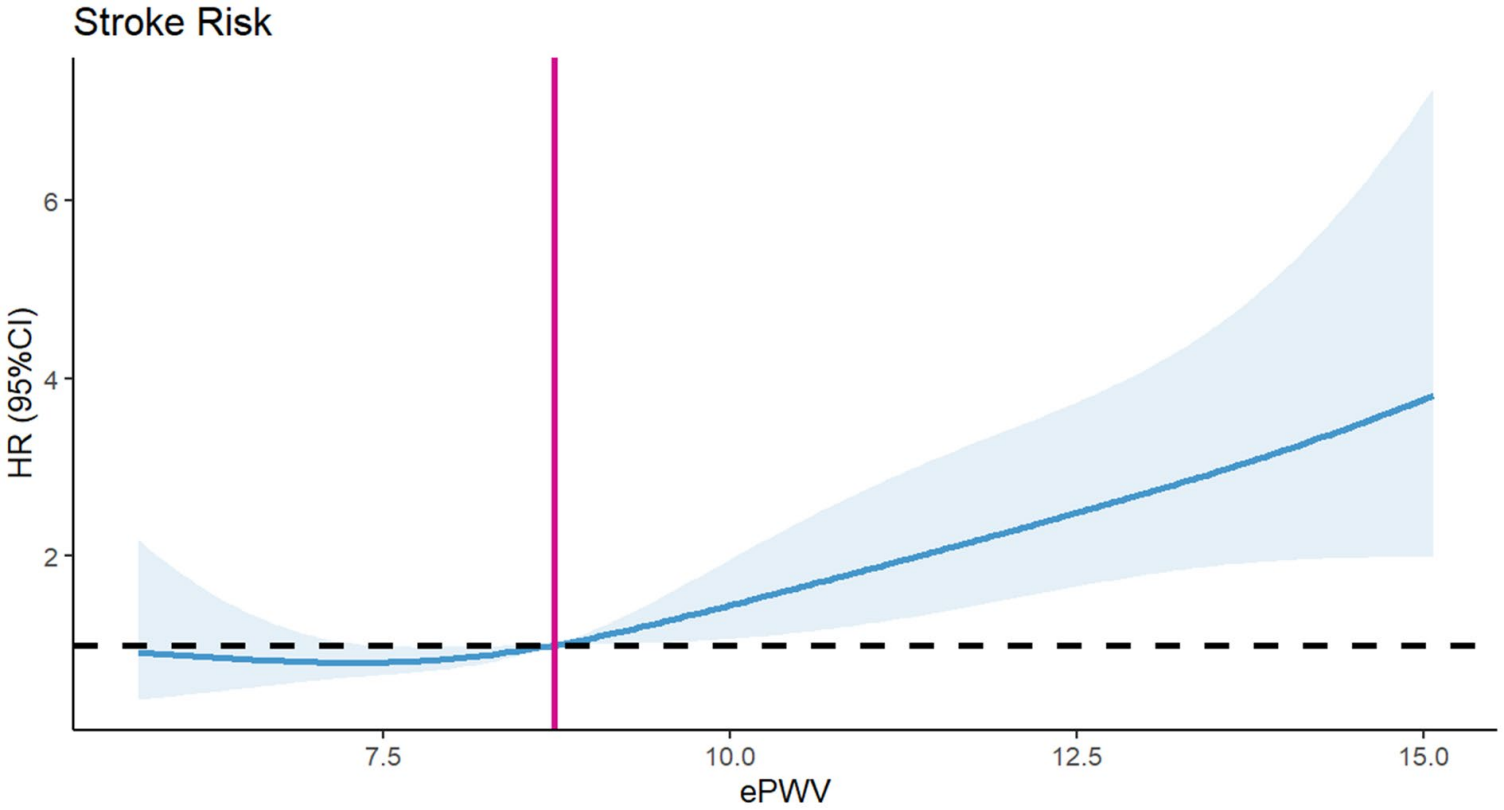
RCS curve for the ePWV hazard ratio and all-cause mortality.

To assess differences in all-cause mortality among different ePWV groups, we generated a Kaplan–Meier survival curve. Analysis of the Kaplan–Meier curve reveals a significantly lower cumulative survival rate among ICU patients with NSAH in the high ePWV group (*p* = 0.00031), as depicted in Figure 2. Even when adjusting for age and sex in Model 1 (HR = 0.5161, *p* = 0.000395), and further covariates in Model 2 (HR = 0.54730, *p* = 0.00937), this correlation persists, with the high ePWV group serving as the reference (Table 2). Subsequent analysis using the RCS model demonstrates an increase in the risk of all-cause mortality with higher ePWV levels (Figure 3). It is noteworthy that this correlation becomes more pronounced when ePWV exceeds 8.728 m/s.

**Table 2:** Cox proportional HR for all-cause mortality.

Group	Non-adjusted Model		Model 1		Model 2	
	HR	*p* value	HR	*p* value	HR	*p* value
High (*n* = 322)	Reference					
Low (*n* = 322)	0.5161	0.000395	0.5522	0.00939	0.54730	0.00937

Non-adjusted Model: unadjusted. Model 1: adjusted for gender and age. Model 2: adjusted for age, gender, MI, CHF, anemia, diabetes, COPD, and AKI.

### Multivariate COX regression analysis and subgroup analysis

**Figure 4:**
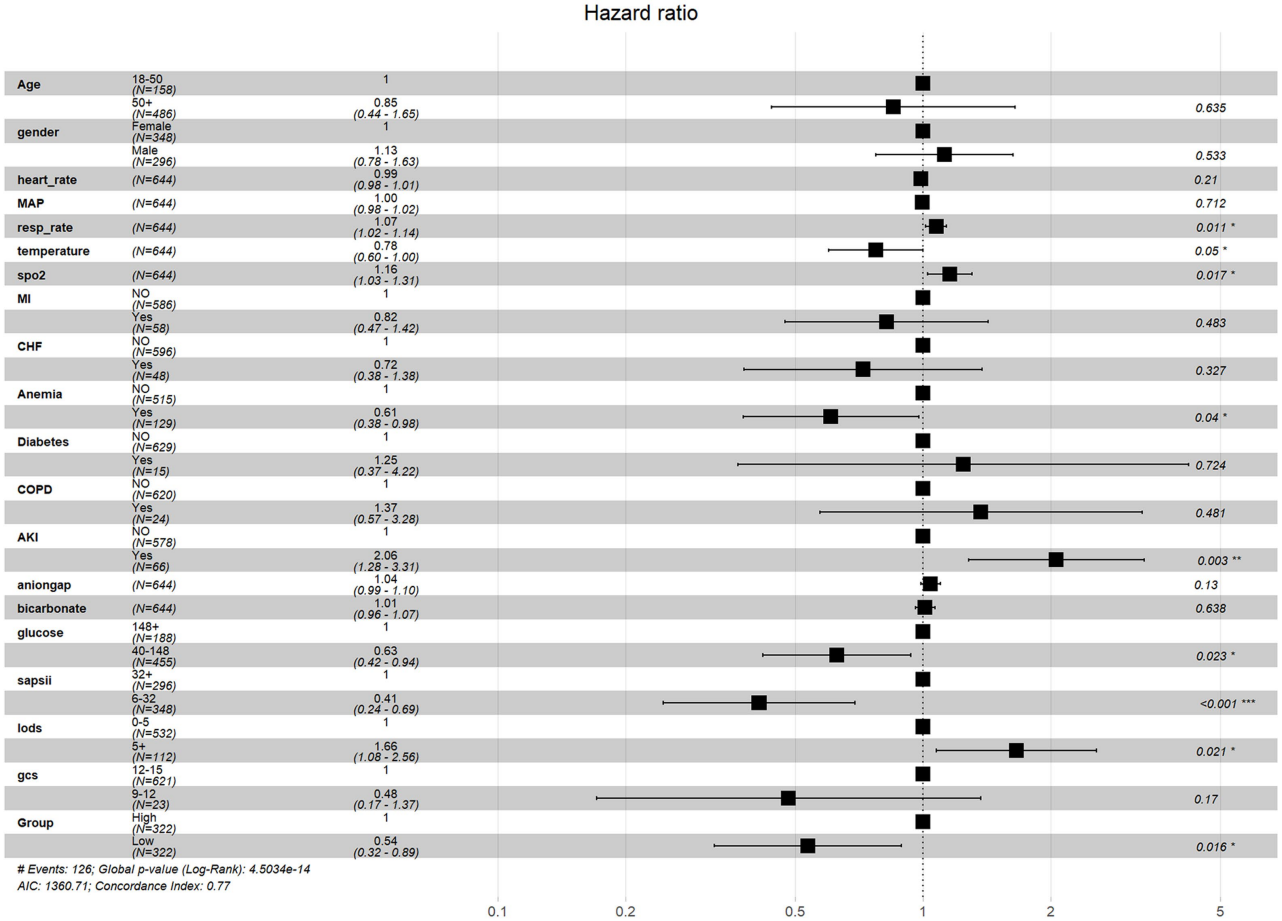
Multivariate cox regression analysis of all-cause mortality risk factors in NSAH patients.

**Figure 5:**
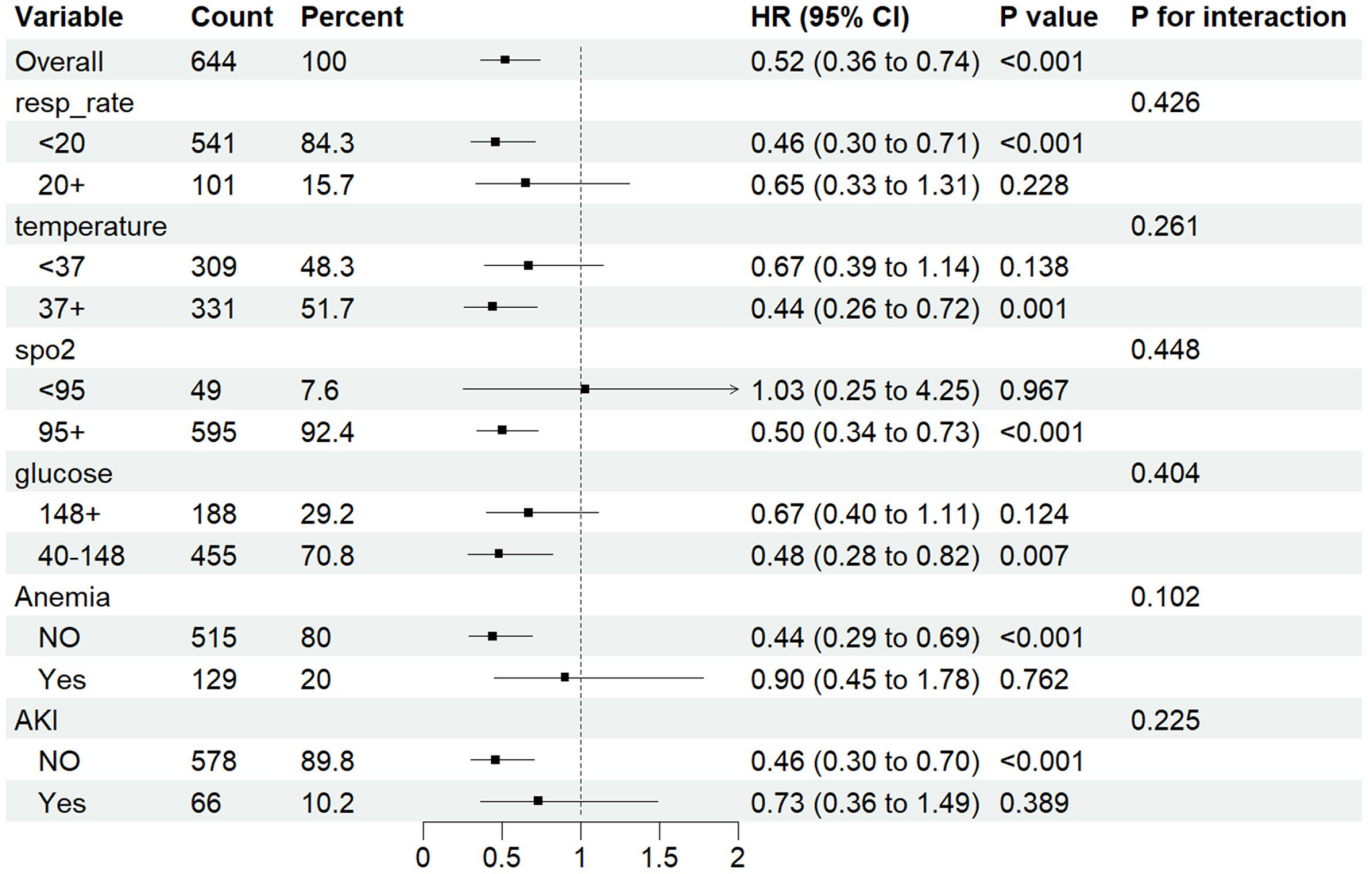
Forest plots for different subgroup analysis of HRs for the association between ePWV and al-cause mortality.

Cox regression analysis revealed ePWV as a significant predictor of all-cause mortality in NSAH patients (GroupLow, HR = 0.54, 95% CI: 0.32–0.89, *p* = 0.016) (Figure 4). Additionally, resp_rate, temperature, spo2, Anemia, AKI and glucose were identified as independent prognostic risk factors. Moreover, we use C-index to evaluate the cox regression model, and finally, C-index = 0.769, which shows that the model has good accuracy. To confirm the association between ePWV and all-cause mortality stratified by resp_rate, temperature, spo2, Anemia, AKI and glucose, a subgroup analysis was performed. As depicted in Figure 5, the findings suggest that ePWV is associated with ICU all-cause mortality in most NSAH patient subgroups. Moreover, no significant interaction was observed between PWV and other subgroups, indicating that the effect of ePWV on mortality was independent of patient characteristics.

## Discussion

This study demonstrates that the ePWV level upon entering the ICU independently predicts the mortality of NSAH patients during hospitalization. Following adjustment for confounding factors through multivariate Cox regression analysis, elevated ePWV remains a significant predictor of all-cause mortality. Specifically, the risk ratio for all-cause death in the ICU among patients with a low ePWV level (≤ 8.728 m/s) is 0.54 times that of patients with a high ePWV level (> 8.728 m/s). Additionally, we observed no interaction between ePWV level and hospital all-cause mortality across various subgroups.

The measurement of carotid-femoral pulse wave velocity (cfPWV) is considered the gold standard for assessing arterial stiffness and vascular aging (6, 16). However, due to technical and procedural challenges, applying cfPWV in daily clinical research is difficult (17).Consequently, some researchers calculate ePWV based on the interaction between actual age and average blood pressure, achieving performance equivalent to real cfPWV measurements, thereby considering ePWV a reliable marker of cardiovascular risk (18, 19). In recent years, some studies have demonstrated that the elevation of cfPWV levels is associated not only with asymptomatic cerebrovascular diseases in hypertensive patients but also with the calcification of intracranial aneurysms in acute stroke patients, affecting the prognosis of patients with intracranial vascular diseases (20, 21). Consistent with these findings, our study demonstrates a significant association between high ePWV levels and increased risk of hospital death.

Our study found that there was a J-type correlation between ePWV at admission and the adverse outcomes of NSAH. When ePWV exceeded 8.728 m/s at admission, the all-cause mortality of patients was significantly higher. Therefore, we suggest that under the condition of ensuring organ perfusion, we can choose a more suitable target blood pressure for patients of different ages by calculating ePWV. However, compared with previous studies, first of all, we excluded patients with more critical illness (GCS<9), mainly considering that the symptoms of cerebral vasospasm and intracranial pressure in these patients may be more serious, thus affecting the regulation of blood pressure. Secondly, we first correlated ePWV with the prognosis of NSAH patients. Thirdly, our research variables took age into account, so that we can determine the optimal blood pressure target value for patients of different ages.

The precise mechanism underlying the close association between ePWV levels and all-cause mortality in NSAH patients remains unclear. Nevertheless, several potential explanations can be proposed. One possibility is that ePWV levels may be associated with systemic inflammation (22), and systemic inflammation following NSAH may play a crucial role in mediating cerebral vasospasm and brain injury (23). Another explanation is that ePWV levels are directly influenced by blood pressure at the time of admission to the ICU. Apart from age, hypertensive patients tend to have elevated ePWV levels, and persistent hypertension is closely associated with the risks of rebleeding, delayed cerebral ischemia, and severe cerebral edema in NSAH patients (24, 25). A third possibility is that the elevation of ePWV may be linked to changes in age-related metabolic symptoms and oxidative stress, with neuronal apoptosis induced by oxidative stress representing one of the significant mechanisms of early brain injury (26, 27). Moreover, high ePWV levels have been associated with obstructive sleep apnea and sympathetic hyperactivity (28). Sleep apnea can exacerbate early brain injury following subarachnoid hemorrhage by exacerbating neuroinflammation and focal death (29). The occurrence of paroxysmal sensory hyperactivity disorder in patients with acute brain injury may lead to extended hospital stays, escalated hospitalization costs, physical impairment, and potential mortality (30).

However, we must acknowledge several limitations of our study. Firstly, the retrospective design may introduce bias in patient selection and analysis. A prospective cohort study is necessary to further investigate the causal relationship between elevated ePWV and NSAH mortality. Secondly, due to limitations in the MIMIC database, crucial data such as mechanical ventilation and vasoactive drug information for NSAH patients were insufficiently represented, precluding their inclusion in the analysis. Thirdly, we limited our data collection to within 6 h prior to ICU admission to assess the association between ePWV levels and mortality. Fourthly, our analysis does not investigate the etiology of NSAH patients or the variation in ePWV levels between the prognoses of aneurysmal and non-aneurysmal NSAH.

## Conclusion

This retrospective study demonstrates that a high ePWV level serves as a significant risk factor for all-cause mortality among NSAH patients in the ICU. By calculating ePWV, clinicians can significantly assist NSAH patients in conducting risk stratification and subsequently developing personalized treatment strategies to enhance patient prognosis.

## Data availability statement

Publicly available datasets were analyzed in this study. This data can be found at: MIMIC-IV 2.2database.

## Ethics statement

The studies involving human participants were examined and approved by Beth Israel Deaconess Medical Center. To protect patient privacy, all data were de-identified; therefore, the Ethical Committee of the Beth Israel Deaconess Medical Center waived the requirement for informed consent. Written informed consent for participation was not required for this study in accordance with the national legislation and the institutional requirements.

## Author contributions

JL: Data curation, Formal analysis, Writing – original draft. MZ: Formal analysis, Funding acquisition, Writing – original draft. BY: Data curation, Formal analysis, Writing – original draft. ML: Methodology, Resources, Writing – original draft. GL: Validation, Visualization, Writing – review & editing.
